# Use of Single-Item Self-Rated Health Measure to Identify Frailty and Geriatric Assessment-Identified Impairments Among Older Adults with Cancer

**DOI:** 10.1093/oncolo/oyab020

**Published:** 2022-01-28

**Authors:** Smith Giri, Nabiel Mir, Mustafa Al-Obaidi, Deanna Clark, Kelly M Kenzik, Andrew McDonald, Crystal Young-Smith, Ravi Paluri, Lakshmin Nandagopal, Olumide Gbolahan, Kirsten A Nyrop, Hyman B Muss, Mackenzi Pergolotti, Smita Bhatia, Grant R Williams

**Affiliations:** 1 Institute for Cancer Outcomes and Survivorship, University of Alabama at Birmingham, AL, USA; 2 Division of Hematology and Oncology, Department of Medicine, University of Alabama at Birmingham, Birmingham, AL, USA; 3 Department of Medicine, University of Alabama at Birmingham, Birmingham, AL, USA; 4 Division of Oncology, The University of North Carolina at Chapel Hill, NC, USA; 5 Revital Cancer Rehabilitation, Select Medical, Mechanicsburg, PA, USA; 6 Department of Occupational Therapy, Colorado State University, Fort Collins, CO, USA

**Keywords:** self-rated health, frailty, geriatric assessment, geriatric oncology, aging

## Abstract

**Background:**

Poor self-rated health (SRH) is a known predictor of frailty and mortality in the general population; however, its role among older adults with cancer is unknown. We evaluated the role of SRH as a potential screening tool to identify frailty and geriatric assessment (GA)-identified impairments.

**Materials and Methods:**

Adults ≥60 years diagnosed with cancer in the UAB *C*ancer & *A*ging *R*esilience *E*valuation (CARE) registry underwent a GA at the time of initial consultation. We measured SRH using a single-item from the Patient-Reported Outcomes Measurement Information System global health scale and dichotomized responses as poor (poor, fair) and good (good, very good, and excellent). We evaluated the diagnostic performance of SRH in measuring frailty, and GA impairment (≥2 deficits among a set of seven GA domains). We examined the impact of SRH with survival using a Cox model adjusting for confounders, exploring the mediating role of frailty.

**Results:**

Six hundred and three older adults with cancer were included, with a median age of 69 years. Overall, 45% (*n* = 274) reported poor SRH. Poor SRH demonstrated high sensitivity and specificity for identifying frailty (85% and 78%, respectively) and GA impairment (75% and 78%, respectively). In a Cox regression model, poor SRH was associated with inferior survival (HR = 2.26; 95% CI 1.60-3.18) after adjusting for confounders; frailty mediated 69% of this observed relationship.

**Conclusion:**

Self-rated health may be used as a screening tool to identify older adults with cancer with frailty and GA impairments. Poor SRH is associated with inferior survival, which is mediated by frailty.

## Teaser

This article evaluates the role of self-rated health as a potential screening tool to identify frailty and geriatric assessment-identified impairments.

**Implications for Practice**

In this prospective cohort study involving 603 older adults ≥60 years with cancer, poor self-rated health at the time of initial consultation with a medical oncologist demonstrated a high sensitivity and specificity for frailty (85% and 78%, respectively) and GA impairment (75% and 78%, respectively), which was independently confirmed in an external replication cohort. Poor SRH was associated with inferior survival after adjusting for age, sex, race, cancer type/stage, and planned therapy. A single item question on self-rated health may be used to screen older adults with cancer who are likely to have frailty and geriatric assessment identified impairments.

## Introduction

More than 60% of new cancer diagnoses occur among adults aged 65 years or older.^1^ Although, older adults with cancer are at high risk of treatment-related toxicity and inferior survival, chronologic age by itself does not adequately explain inter-individual variability in outcomes.^2^ A geriatric assessment (GA) can identify vulnerable or “frail” older adults at greatest risk for treatment-related adverse events^3–5^, health care utilization,^4^ and mortality^6^. Currently, the American Society of Clinical Oncology^7^ and the National Comprehensive Cancer Network (NCCN)^8^ recommend using GA to personalize and guide clinical care in older adults. Yet, widespread use of GA in oncology practice remains limited due to perceived complexity as well as time/resource constraints.^9^ While there are few screening tools to identify patients at greatest risk of GA impairments,^10,11^ a primarily self-administered single-item screening tool represents an unmet need.

Self-rated health (SRH) is a widely used measure of health, where patients are asked to report their health status based on their own definition of health.^12^ It is typically measured using a *single item question*, commonly worded as “In general would you say your health is” with the response items “excellent”, “very good”, “good”, “fair” or “poor”. While, SRH captures an individual’s perception of their overall health, it has been shown to correlate well with performance-based measures of health.^13^ Among community-dwelling older adults, SRH predicts functional limitation, cognitive impairment, health care utilization, and survival.^14–17^ Yet, there is limited data regarding the utility of this tool to predict adverse outcomes among older patients with cancer.

Here, we evaluated SRH as a screening tool to identify older adults with cancer at risk of frailty and GA-identified impairments and its association with mortality.

## Methods

### Study Population

We used participants from the University of Alabama at Birmingham (UAB) Cancer and Aging Resilience Evaluation (CARE) study—a prospective registry enrolling older adults (≥60 years) undergoing cancer treatment at UAB Hospitals and Clinics.^18,19^ We chose 60 years of age as criteria for enrollment in this registry given recognition of the uncertainty of the “right” age cutoff and to allow for meaningful age-related sub-analyses such as the current study.^20^ For the current report, we included patients completing GA at the time of initial consultation to the UAB medical oncology clinic between 9/2017 and 10/2019. The Institutional Review Board of UAB (IRB-300000092) approved this study.

### Self-Rated Health

We measured SRH using a single-item question from the Patient-Reported Outcomes Measurement Information System (PROMIS) global health scale, incorporated as a part of the GA.^21^ The question is worded as: “In general, would you say your health is?” with the following possible responses scaled on a 5-point Likert scale, “excellent”, “very good”, “good”, “fair”, or “poor”. We then dichotomized these responses to poor (poor, fair) and good (good, very good, excellent), consistent with other studies.^22,23^

### Baseline Geriatric Assessment

All participants completed patient-reported GA as previously published (Supplementary Table S1).^18,19^ Briefly, CARE GA is a modified version of the Cancer and Aging Research Group GA^24^ and includes the following domains^25,26^: functional status,^27,28^ comorbidity,^27,29,30^ cognition, mood disorders,^31–33^ nutrition,^34^ social support,^35^ and HRQoL^33^, consistent with recommendations from the International Society of Geriatric Oncology.^25,26^

### Frailty Index

We constructed a frailty index using the principle of deficit accumulation approach originally described by Rockwood et al,^36^ and following the standard procedures outlined by Searle et al^37^ We used 44 GA variables from the CARE survey (Supplementary), and categorized patients as robust (0-0.2), pre-frail (0.2-0.35), and frail (>0.35), as previously described.^37^ An index constructed with ≥30 items has been previously shown to predict adverse outcomes and survival among older adults.^38^

### GA Impairment

We classified patients as having significant GA impairment if they had ≥2 impairments among the following seven domains^10,11,39^: impairment in any activities of daily living (ADL); impairments in ≥2 instrumental activities of daily living (IADL); limitation in walking one block; significant weight loss (3% in 3 months or 6% in 6 months); presence of ≥4 comorbidities; presence of depression (*T*-score ≥60) and cognitive impairment (*T*-score ≤40) (Supplementary). Additionally, we conducted a sensitivity analysis varying our definition of GA impairment as ≥1 GA impairment as well as ≥3 GA impairments.

### Additional Variables

Information on vital status was updated to January 12, 2019, linking the study cohort to Accurint database,^40^ which uses death information from Social Security Administration records, obituaries and state death records, and supplemented by review of medical records. We abstracted information on demographic and clinical characteristics including age at the time of GA evaluation, sex, race/ethnicity, cancer type, cancer stage, and planned systemic therapy from medical records.

### External Validation

We obtained data on 372 older adults ≥60 years with cancer enrolled in the Carolina Senior Registry (*n* = 208) and four exercise intervention trials (*n* = 164; NCT011789983, NCT02167932, NCT02328313, and NCT037611706) at the University of North Carolina at Chapel Hill.^41^ We measured and similarly categorized SRH based on a single-item question from the PROMIS global health scale. Frailty was defined using the 36-item Carolina Frailty Index as previously reported.^6^ Meanwhile, GA impairment was defined as the presence of ≥2 impairments among the following seven domains: impairments in ≥2 IADL; abnormal timed get up and go test (≥20 s^42^); limitation in walking one block; significant weight loss (5% within 6 months); presence of ≥4 comorbidities using OARS comorbidity assessment; presence of depression (13-item Mental Health Index score ≥12^43^); and cognitive impairment (Blessed Orientation Memory Concentration test score ≥11^44^). Of note, data on ADL were not available and abnormal TUG ≥20 s was used as a surrogate for ADL, as shown before.^10,45^

### Statistical Analyses

We compared the baseline demographic, clinical and GA characteristics between patients reporting poor and good SRH by using distribution appropriate statistical tests. We examined the diagnostic performance of SRH for measuring frailty (frail vs others) and GA impairment in terms of sensitivity, specificity, positive predictive value, negative predictive value, and receiver operating characteristic curve (ROC AUC). We computed binomial exact 95% confidence intervals for sensitivity, specificity, positive predictive value, and negative predictive value. Meanwhile, the AUC and its asymptotic normal 95% confidence interval was calculated using the non-parametric method proposed by DeLong et al^46^ We identified the optimal cut point for dichotomizing original SRH responses using the method proposed by Liu^47^, aiming to maximize the product of sensitivity and specificity. Additionally, we reported the above diagnostic performance, by varying our definitions of GA impairment as presence of ≥1, or ≥3 GA domain impairments, as well by restricting to those age ≥70 years. We then independently confirmed our findings in the replication cohort.

The median follow-up of the study cohort was estimated using reverse Kaplan–Meier method.^48^ To account for variation in time between the date of diagnosis and time of GA evaluation, we used a landmark analysis using the date of GA evaluation as the start of follow-up. We used Kaplan–Meier methods and log-rank test to compare the survival distributions between those reporting good vs poor SRH. We used Cox proportional hazards regression to evaluate the independent impact of SRH on overall survival adjusting for potential confounders including age, sex, race, cancer type/stage, and planned systemic therapy. We tested proportionality assumption of the Cox model using Schoenfeld residuals and testing for interaction using log person-time; covariates violating this assumption were used as stratification variables in the final model.

Finally, we hypothesized that frailty might act as a mediator between SRH and survival. Conceptually, a mediation analysis attempts to break down the total effect of an exposure (SRH) on outcome (overall survival) into two parts: (1) direct effect: effect of an exposure on an outcome in the absence of mediators and (2) indirect effect: effect of an exposure on outcome explained by a set of mediator (Frailty). To allow for interaction between SRH and frailty, we used the four-way decomposition as proposed by Vanderweele to conduct this mediation analysis. This method breaks down the overall effect (the total effect) of an exposure (self-rated health) on outcome (overall survival), in the presence of a mediator (frailty) into four components; (1) the effect of exposure in the absence of the mediator, (2) effect due to interaction only, (3) effect due mediation and interaction, and (4) due to mediation only.^49^ We estimated mediation with “exposure” defined as a dichotomized measure of self-reported health (poor/fair vs. good/excellent) and the mediator focused on frailty index as a continuous variable. We specified an accelerated failure time model with Weibull distribution and used the Med4way package in STATA^50^ to estimate the causal contrasts that arise in the above decomposition.

All hypothesis testing was two-sided, and the level of the significance was set at .05. We conducted statistical analysis using SAS statistical software version 9.4 (SAS Institute Inc., Cary, NC) and STATA v13.0 (StataCorp LLC, College Station, TX).

## Results

Of the 708 older adults diagnosed with cancer in the CARE registry, 603 (85%) underwent GA and reported on SRH at the time of initial consultation with a medical oncologist (Supplementary Figure S1). The median time from diagnosis to GA was 37 days (IQR 19-112 days). The median age at study enrollment was 69y (IQR 64-274 years), with 61% females and 75% whites. Common cancers included colorectal cancer (24%), pancreatic cancer (17%), and hepatobiliary cancers (13%). Almost half of the participants presented with stage IV disease (Table 1).

**Table 1.** Comparison of baseline demographic and clinical characteristics among patients reporting poor and good self-rated health.

Variable	Poor SRH	Good SRH	*P*-value
N	274	329	
Demographic characteristics			
Age in years, median (IQR)	68 (63-74)	69 (64-75)	.02
Age category, *n* (%)			.13
60-69 years	162 (59.1%)	165 (50.2%)	
70-79 years	84 (30.7%)	129 (39.2%)	
80-89 years	26 (9.5%)	33 (10%)	
90+	2 (0.7%)	2 (0.6%)	
Sex, *n* (%)			.02
Male	94 (34.3%)	143 (43.5%)	
Female	180 (65.7%)	186 (56.5%)	
Race, *n* (%)			.09
White	195 (71.2%)	254 (77.2%)	
Black	76 (27.7%)	68 (20.7%)	
Other	3 (1.1%)	7 (2.1%)	
Marital status, *n* (%)			.19
Single	23 (8.4%)	17 (5.2%)	
Widowed/divorced/separated	73 (26.6%)	86 (26.1%)	
Married	165 (60.2%)	217 (66.0%)	
Unknown	13 (4.7%)	9 (2.7%)	
Education, *n* (%)			<.001
Less than high school	53 (19.3%)	41 (12.5%)	
High school graduate	82 (29.9%)	69 (21.0%)	
Associate/bachelors	106 (38.7%)	173 (52.6%)	
Advanced degree	15 (5.5%)	41 (12.5%)	
Unknown	18 (6.6%)	5 (1.5%)	
Clinical characteristics			
Time from diagnosis, median (IQR) days	38 (19, 120)	37 (18, 112)	.95
Cancer type, *n* (%)			.04
Colorectal	54 (19.7%)	93 (28.3%)	
Pancreatic	58 (21.2%)	46 (14.0%)	
Hepatobiliary	33 (12.0%)	45 (13.7%)	
Gastroesophageal	25 (9.1%)	21 (6.4%)	
Neuroendocrine carcinoma	19 (6.9%)	19 (5.8%)	
Prostate cancer	17 (6.2%)	26 (7.9%)	
Lung cancer	22 (8.0%)	14 (4.3%)	
Head and neck cancer	12 (4.4%)	20 (6.1%)	
Other	34 (12.4%)	45 (13.7%)	
Cancer stage, *n* (%)			.03
Stages I-II	65 (23.7%)	85 (25.8%)	
Stage III	52 (36.1%)	92 (28.0%)	
Stage IV	151 (50.7%)	147 (44.7%)	
Unknown	6 (54.6%)	5 (45.5%)	
Planned chemotherapy, *n* (%)			.02
No	71 (25.9%)	60 (18.2%)	
Yes	203 (74.1%)	269 (81.8%)	

Abbreviations: SRH, self-rated health; IQR, inter-quartile range; CARE, Cancer and Aging Resilience Evaluation.

Most patients responded to the single item SRH question as good (33.7%; *n* = 203) or fair (31.5%; *n* = 190) (Supplementary Figure S2). Upon dichotomizing the responses as poor (poor and fair) or good (good, very good, and excellent), poor SRH was reported by 45% of participants (*n* = 274). When compared with patients with good SRH, those reporting poor SRH were more likely to be female (66% vs 57%; *P* = .02), have less than high school education (19% vs 13%, *P* < .001), diagnosis of pancreatic cancer (21% vs 14%; *P* = .04), and stage IV disease (56% vs 45%; *P* = .01) (Table 1).

### Association of SRH with Frailty and Geriatric Impairment

**Figure 1.**
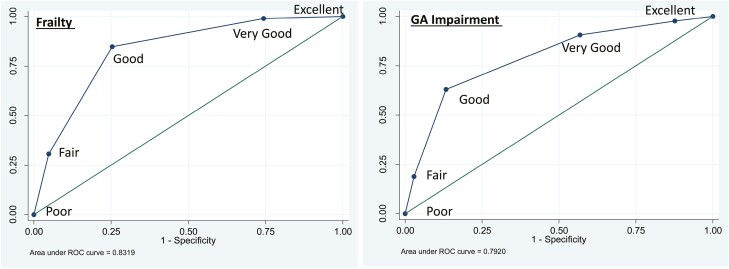
Comparison of Geriatric Assessment findings among patients with poor and good self-rated health. Height of the bar represent proportion of patients in each category. When compared with patients with good SRH, those reporting poor SRH had significantly higher prevalence of frailty and GA impairments in several domains.

The prevalence of frailty was significantly higher among patients with poor SRH when compared with those reporting good SRH (68% vs 10%; *P* < .001). Similarly, patients with poor SRH were more likely to have GA impairment (≥2 GA domain impairments) When compared with those with good SRH (74% vs 21%, *P* < .001) as well as domain-specific impairments in ADL, IADL, depression anxiety, multi-morbidity, polypharmacy, malnutrition, and one or more falls (Figure 1).

### Diagnostic Validity of SRH as a Screening Tool for Frailty and GA Impairments

We first confirmed that our binary classification of SRH, ie, good (good, very good, and excellent) versus poor (poor or fair) was also the optimal cut point offering the best balance between sensitivity and specificity (Figure 1). Using this cut point, SRH demonstrated high sensitivity (85%) and specificity (78%) for identifying frail patients, with a positive predictive value (PPV) of 69% and negative predictive value (NPV) of 90%. Similarly, the sensitivity and specificity of SRH in identifying patients with GA impairment (≥2 GA domain impairments) was 75% and 78%, respectively, with a PPV of 74% and NPV of 79% (Table 2). Finally, in a pre-planned sensitivity analysis, the sensitivity/specificity of SRH in identifying patients with ≥1 GA domain impairment and ≥3 GA domain impairment was 58/87% and 81/69%, respectively. Similarly, consistent results were obtained when limiting our cohort to age ≥70 years (Supplementary Table S2).

**Table 2.** Diagnostic performance of self rated health in identifying impairment compared with the comprehensive geriatric assessment and frailty using cumulative deficit frailty index.

Reference standard	% Impaired	Sensitivity (95% CI)	Specificity (95% CI)	PPV	NPV	AUC (95% CI)
Derivation cohort (UAB CARE Registry)						
Frail	36.8%	84.9% (79.4-89.3%)	78.4% (73.9-82.4)	69.6% (64.6-75.0%)	89.9% (86.1-92.9%)	0.82 (0.78-0.85)
≥2 GA impairment	45.2%	74.5% (69.3-79.7%)	78.4% (74.0-82.7%)	74.0% (68.2-79.1%)	78.9% (74.0-83.2%)	0.76 (0.73-0.80)
Replication cohort (University of North Carolina)						
Frail	16.5%	60.7%	82%	40.0%	91.4%	0.71 (0.65-0.78)
≥2 GA impairment	25.8%	60.5% (49.3-70.8%)	86.7% (81.8-90.7%)	61.2% (49.9-71.6%)	86.4% (81.4-90.4%)	0.74 (0.68-0.79)

Abbreviations: GA, geriatric assessment; PPV, positive predictive value; NPV, negative predictive value; AUC, area under the curve; CI, confidence interval.

### Association of SRH with Mortality

**Figure 2.**
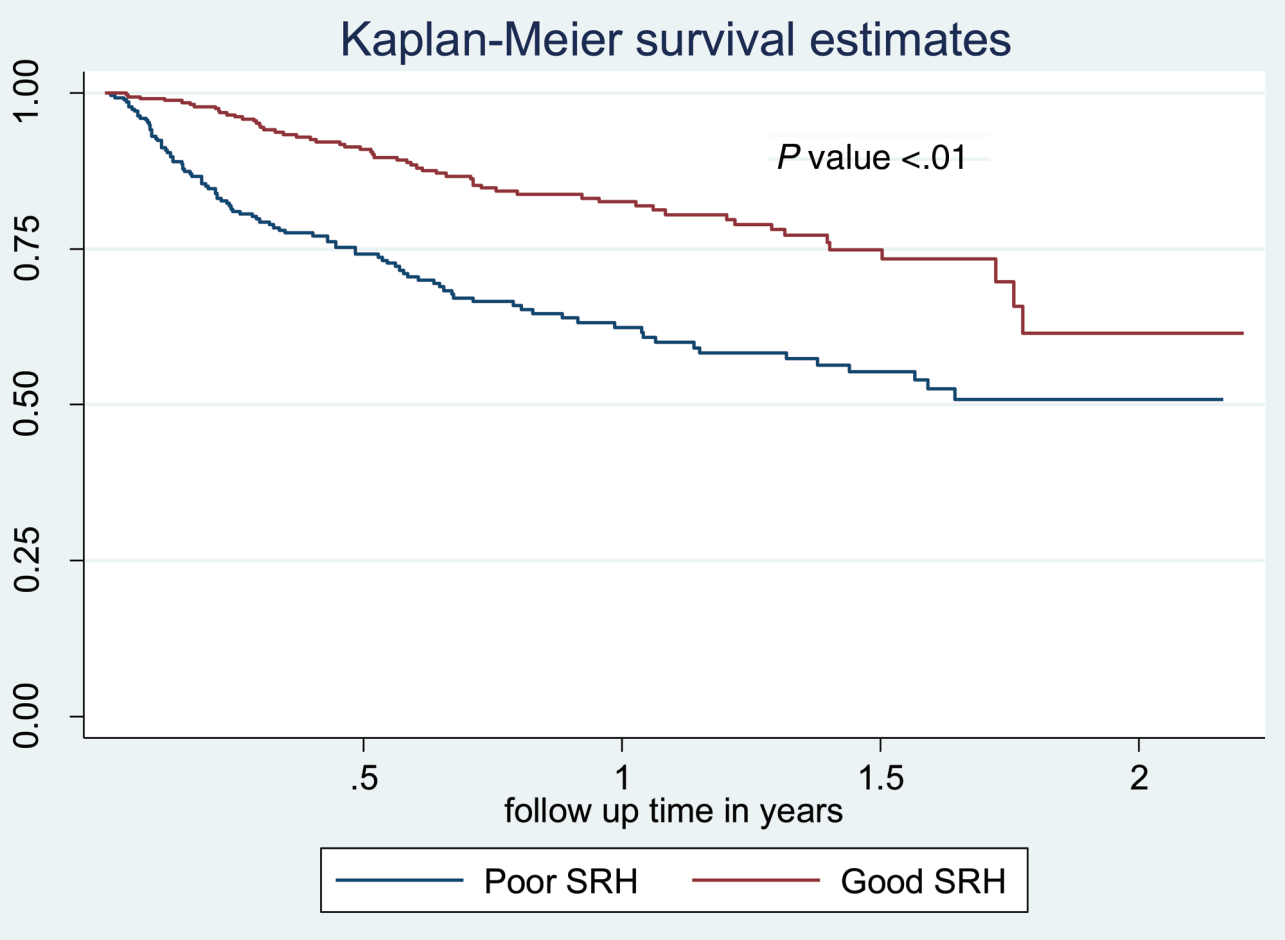
Kaplan–Meier curve showing the comparison of survival distribution between patients with good or poor SRH. When compared with patients with good SRH, those reporting poor SRH had a significantly worse survival (1-year OS 62.4% vs 82.6%, log-rank *P-*value < .001).

When compared with patients with good SRH, those reporting poor SRH had a significantly worse survival (1-year OS 62.4% vs 82.6%, log-rank *P <* .001; Figure 2). In a multivariable Cox regression analysis, poor SRH remained an independent predictor of worse survival (hazard ratio [HR] 2.26 [1.60-3.18], *P* < .001) after adjusting for age, sex, race, cancer type, and planned chemotherapy as well as accounting for cancer stage as stratification variable (Table 3).

**Table 3.** Multivariable cox proportional hazards regression model showing association between self-rated health and overall survival^a^.

Variable	Hazard ratio	95% CI	*P*-value
Self-rated health			<.001
Good	Ref	—	
Poor	2.26	1.60-3.18	
Age in years (continuous)	0.99	0.98-1.02	.99
Sex			.78
Female	Ref	—	
Male	1.05	0.74-1.48	
Race			
White	Ref	-	
Black	0.83	0.56-1.24	.36
Other	2.48	0.75-8.16	.14
Planned chemotherapy	0.90	0.60-1.34	.61
Cancer stage			
Stages I-II	Ref	—	
Stage III	0.81	0.47-1.41	.46
Stage IV	2.43	1.61-3.69	<.001

Cancer type was treated as a stratification variable in the survival analysis.

Index date for survival analysis is the date of CARE survey including SRH assessment.

### Association of Frailty with Mortality

We confirmed that the CARE-Frailty Index provided stratification of survival, discerning groups with 1-year overall survival ranging between 85.7% for robust, 76.5% for pre-frail, and 58.5% for frail patients, respectively (log-rank test for trend *P* < .001) (Supplemantary Figure S2). Furthermore, in a multivariable Cox regression model, frail patients had a worse overall survival when compared to non-frail patients (HR 2.25 [1.27-3.98], *P <* .001) after adjusting for age, sex, race, cancer type, cancer stage, SRH, and planned chemotherapy. Interestingly, the prognostic impact of SRH remained no longer significant (HR 1.44 [0.91-2.29], *P* = .12) after adding frailty into the multivariable analysis, suggesting a possible mediating role (Supplemantary Table S3).

### Mediation Analyses

We conducted a four-way decomposition analysis to identify the proportion of the observed relationship between SRH and mortality (ie, total effect) that is mediated by frailty. The details of the mediation analysis are presented in supplementary files (Supplemantary S1; Table S4), where the effect sizes of each component are represented in the ratio scale and the total and the total excess risk is broken down into four parts: (1) excess relative risk due controlled direct effect (effect of SRH on survival without mediation or interaction), (2) excess relative risk due to reference interaction (interaction only), (3) excess relative risk due to mediated interaction (due to both interaction and mediation), and (4) excess relative risk due to pure indirect effect (due to mediation only). Based on our four-way decomposition analysis, we found that frailty as a mediator explained 69% of the observed impact of SRH on survival, whereas, the direct effect and interaction terms were not significant (Supplemantary Table S4).

### External Validation

Our replication cohort comprised of 372 older adults ≥60 years with cancer, with a median age of 73 years (range 60-91 years), 47% females, 86% non-Hispanic white and with multiple myeloma (18%), breast cancer (17%), and prostate cancer (13%) being most common diagnoses. In this cohort, the prevalence of frailty was 17%, whereas 26% had significant GA impairment (≥2 GA domain impairments). Self-rated health demonstrated moderate sensitivity (61%) and high specificity (82%) for capturing frailty, as well as GA impairment (61% sensitivity, 87% specificity) (Table 2).

## Discussion

In this study of 975 older adults with cancer, we found that a single-item SRH question has a moderate-to-high sensitivity and a high specificity for capturing frailty and GA impairment, suggesting its value as a simple, one-item screening tool for identifying vulnerable older adults with cancer who require further evaluation with a GA. Furthermore, we demonstrated that poor SRH is independently associated with inferior survival, and this effect is largely mediated by a higher prevalence of frailty in this population.

Despite overwhelming evidence regarding the role of GA in capturing frailty and GA impairments among older adults with cancer, widespread implementation remains limited due to perceived complexity, interruption in clinic workflow, and time/resource constraints.^51^ To our knowledge, this is the first study to demonstrate the utility of single-item SRH question as a potential screening tool for identifying older cancer patients with frailty and GA impairments. Among older adults ≥60 years with chronic kidney disease, single item SRH demonstrated a 70.0% sensitivity and 92.0% NPV in identifying frail individuals using Fried phenotype as well as Dalhousie Clinical Frailty Score^52^. Similar findings were reported in 243 community dwelling older adults aged ≥75 years, where SRH had a sensitivity of 62.5%, specificity of 93.6%, and a PPV/NPV of 67.5%/92.2%^53^. Notably, both studies report a high NPV of this tool (≥90%), arguing that patients reporting excellent, very good or good SRH are very unlikely to have frailty and a comprehensive GA can be likely deferred.

We found that SRH was associated with several domain-specific impairments among older adults with cancer. This is consistent with prior observations that older adults reporting poor SRH have a higher prevalence of ADL impairments,^54^ IADL impairments,^55^ cognitive impairment,^56,57^ malnutrition,^57^ and multimorbidity^57^. Additionally, SRH independently predicted overall survival in our cohort; a finding that has been reported among community dwelling older adults,^14^ older adults presenting to the ED,^54^ or hospital,^58^ and those with chronic kidney disease,^59^ coronary artery disease,^59^ and cognitive impairment.^56^ Only few studies, however, have evaluated the association of SRH with mortality among patients with cancer^60–63^; among 735 adults with advanced malignancy, SRH predicted survival independent of the individual’s performance status.^61^ In another population-based observational study, SRH status predicted non-prostate cancer mortality among men with localized prostate cancer.^62^ Furthermore, we show that this association is mediated by frailty, demonstrating the value of SRH in capturing frailty among older adults with cancer.

Recognizing the challenge to identify older adults with cancer for whom a comprehensive GA would be most beneficial, several screening tools have been previously explored. Bellera et al evaluated the G-8 tool, incorporating age and seven items from the Mini Nutritional Assessment and reported a sensitivity of 85% and 92% as well as specificity of 65% and 48% respectively for identifying patients with ≥1 and ≥2 GA impairments, respectively.^10^ Similarly, the Flemish version of the Triage Risk Screening Tool (fTRST), a five-item screening tool, had a sensitivity of 67% and specificity of 74% in identifying older adults ≥70 years with cancer who had ≥2 geriatric impairments.^39^ Finally, among older adults ≥70 years with prostate cancer, Mohile et al showed that the 13-item Vulnerable Elders Survey (VES-13) had a sensitivity of 73% and specificity of 86% in identifying older adults with ≥2 GA impairments.^11^ It is important to note that both G-8 and VES-13 include a self-rated health as a component. However, in contrast to the above screening tools, SRH is a simple, practical, and an easily scalable single-item screening tool. Moreover, we show for the first time that SRH can identify frail older adults with cancer as well.

Our study has limitations. Over 60% of our cohort had gastrointestinal malignancies recruited from a single institution in the southeastern USA. As such, our findings may not be readily applicable to other malignancies or populations. Of note, our replication cohort from a different site with different cancer types had similar results. We cannot rule out the possibility of selection bias, where fit patients with good health may be more likely to complete the CARE survey as opposed to those in poor health. However, over 92% of all newly diagnosed gastrointestinal malignancies presenting to the GI oncology clinic are currently included in the CARE registry.^2^ We had limited clinical information regarding type and intensity of chemotherapy, tumor grade, lymph node involvement, and tumor markers that may adversely affect survival in our cohort. Our follow-up time was relatively short; additional follow-up with more mature survival data is needed. We were not able to directly compare SRH with established GA screening tools such as G-8,^10^ fTRST,^64^ and VES-13^65^. Finally, due to the self-reported nature of this instrument, SRH may not be useful as a screening tool for older adults with moderate to severe cognitive impairment.

In conclusion, a single-item SRH question is a potential screening tool for capturing frailty and GA impairments among older adults with cancer. Future studies should validate our findings in geographically diverse cohorts across different cancer types and compare it against established screening tools.

## Supplementary Material

oyab020_suppl_Supplementary_Material

## Data Availability

The data underlying this article will be shared on reasonable request to the corresponding author.
