# Supplementary material for: Use of Single-Item Self-Rated Health Measure to Identify Frailty and Geriatric Assessment-Identified Impairments Among Older Adults with Cancer
Source: Oncologist. 2022 Jan 28;27(1):e45–52. doi: 10.1093/oncolo/oyab020 (PMC8842332; doi:10.1093/oncolo/oyab020)
Supplement: oyab020_suppl_Supplementary_Material [file oyab020_suppl_supplementary_material.docx]

**Use of Single-Item Self-Rated Health Measure to Identify Frailty and Geriatric Assessment-Identified Impairments Among Older Adults with Cancer**

**SUPPLEMENTARY FILE**

**Contents:**

1. **Overview of geriatric assessment measures by domain included in CARE survey…………...… 2**
2. **Flow Chart showing Cohort selection ……………………………………………………………...…….3**
3. **Definition of geriatric assessment identified impairments……………………..…………………….. 4**
4. **Construction of CARE frailty index………………………………………………………………………5-6**
5. **Original response to single-item self-rated health question in our study cohort.…………………7**
6. **Receiver operating characteristic (ROC) curve for self-reported health for the diagnosis of frailty (panel A) and geriatric assessment (GA) identified impairments (panel B).……..…………8**
7. **Kaplan Meier plot showing the impact of frailty category on survival ………………………...…... 9**
8. **Cox model showing predictors of Overall Survival after adjusting for Frailty…………………... 10**
9. **Sensitivity Analysis for evaluating Diagnostic Performance of Self Rated Health (SRH) for identifying GA impairment with varying cutoffs …………………………………………………….…11**
10. **Mediation Analysis methodology and main results …………………………………………………..12**
11. **References ……………………………………………………………………………………………... 12-13**

| **eTable 1. Overview of Geriatric Assessment Measures by Domain** | |
| --- | --- |
| **GA Domain** | **Patient-Reported Measures** |
| **FUNCTION** | OARS Instrumental Activities of Daily Living (IADL)^1^  OARS Activities of Daily Living (ADL)^1^  Patient-reported ECOG Performance Status^2^  No. of falls in last 6 months^3^ |
| **NUTRITION** | Patient-Generated Subjective Global Assessment^2,4^ |
| **COGNITION** | PROMIS Cognitive Function^5^ |
| **PSYCHOLOGICAL** | PROMIS Anxiety^6,7^  PROMIS Depression^6,7^ |
| **SOCIAL SUPPORT** | MOS Social Support Survey^8^  (Emotional/Informational Support subscales) |
| **COMORBIDITY** | No. of medications^9^  OARS comorbidity assessment^1,10^  Vision and Hearing Assessment |
| **HRQOL** | PROMIS 10-item Global Health^11^ |

**Abbreviations:** GA, Geriatric Assessment; OARS, Older American Resources and Services; ECOG, Eastern Cooperative Oncology Group; PROMIS, patient-reported outcomes measurement information system; MOS, medical outcomes survey; HRQOL, health-related quality of life


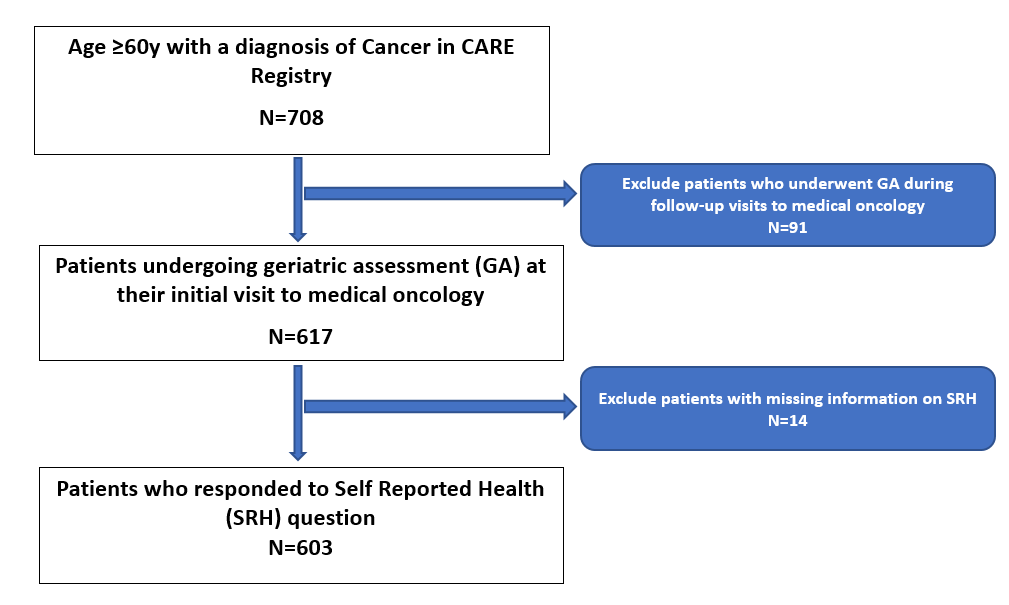


**eFigure 1:** Flow Chart showing the process of study cohort selection. A total of 708 patients were identified in the registry, after excluding 91 patients who underwent geriatric assessment during their follow up/return visits and 14 patients with missing data on self-rated health, a total of 603 patients were included in the final analysis.

**Definition of Global GA Impairment**

Global GA impairment was defined as presence of two or more of the following seven domain specific impairments, consistent with prior studies. Patients without global GA impairment (score 0 or 1) had to have complete information for no less than 6 items, otherwise this measure was treated as missing.

1. Presence of any impairment in Older Americans Resources and Services Scale (OARS) Activities of Daily Living (ADL)
2. Presence of two or more impairments in OARS Instrumental Activities of Daily Living (IADL).
3. Significant limitations in walking one block. Patients responding to the question “Does your health limit you in walking one block?” as “limited a lot”.
4. Presence of significant weight loss. This is defined as (3% weight loss within 3 months or 6% weight loss within 6 months)
5. Multimorbidity defined as presence of four or more comorbidities in the OARS comorbidity assessment.
6. Depression defined as PROMIS Depression T score >60
7. Cognitive impairment, defined as PROMIS Cognitive function T score <40.

**Construction of CARE Frailty Index:**

We constructed a frailty index (hereafter known as the CARE Frailty Index) using the deficit accumulation approach originally described by Rockwood et al^12^, and following the standard procedures outlined by Searle et al^13^. Similar methods have been used by Guerard et al^14^ and Cohen et al^15^ to construct frailty indices that have been shown to be predictive of chemotherapy toxicity and drug discontinuation^15^ as well as all-cause mortality^14^ among older adults with cancer. We selected 44 GA variables from the CARE survey, each of which captured a health deficit, and recoded responses as ‘0’ for absence of the deficit and ‘1’ for presence of the deficit. For variables that included a single intermediate response (e.g. ‘sometimes’ or ‘maybe’), we used an additional value of ‘0.5’. We combined the 44 individual scores into an aggregate frailty score reflecting the overall proportion of deficits (range 0-1), and then categorized patients as robust (0-0.2), pre-frail (0.2-0.35) or frail (>0.35), as previously described.^13^ In case of missing data, we required responses to at least 30 items to construct a valid frailty index. An index constructed with at least 30 variables has been previously shown to be sufficiently accurate for predicting adverse outcomes among older adults.^16^ The 44 variables used for construction of CARE-frailty index are as below.

1. Falls ≥1, 1 point
2. Walk one block = limited a lot , 1 point
3. IADL mobility (unable to/ with some help) , 1 point
4. IADL shopping (unable to/ with some help) , 1 point
5. IADL meal prepare ( unable to/ with some help) , 1 point
6. IADL housework ( unable to/ with some help) , 1 point
7. IADL medication ( unable to/ with some help) , 1 point
8. IADL money ( unable to/ with some help) , 1 point
9. ADL get in and out of bed ( unable to/ with some help) , 1 point
10. ADL dress ( unable to/ with some help) , 1 point
11. ADL bath ( unable to/ with some help) , 1 point
12. Global health, good =0.5 point, fair/poor = 1 point
13. Global quality of life, good =0.5 point, fair/poor = 1 point
14. Global physical health, good =0.5 point, fair/poor = 1 point
15. Global mental health, good =0.5 point, fair/poor = 1 point
16. Global satisfaction with social activities and relationship, good =0.5 point, fair/poor = 1 point
17. Global everyday activities, moderately=0.5 point, a little/not at all = 1 point
18. Global anxious/depression, sometimes =0.5 point, often/always = 1 point
19. Global fatigue, moderate =0.5 point, severe/very severe = 1 point
20. Global pain, pain level 4-6 =0.5 point, pain level 7-10 = 1 point
21. Global social activities and roles, good =0.5 point, fair/poor = 1 point
22. Weight loss 3 months or 6 months’ weight loss >=5%, 1 point
23. Food intake less than usual, 1 point
24. Activities and function (self-rated activity) ≥2 (in bed or chair less than half the day/ able to do little activity / Pretty much bedridden) , 1 point
25. Anxiety PROMIS T score >60, 1 point
26. Depression PROMIS T score >60, 1 point
27. Impaired Cognition, PROMIS T score <40, 1 point
28. Number of daily medication ≥9, 1 point
29. Social activity interference, Some of the time=0.5 point, Most/ All of the time=1 point

**Comorbidities:**

1. Eyesight Fair/Poor/totally blind, 1 point
2. Hearing fair/Poor/Totally Deaf, 1 point
3. Other Cancers or leukemia, 1 point
4. Arthritis or rheumatism, 1 point
5. Glaucoma, 1 point
6. Emphysema or chronic bronchitis, 1 point
7. High blood pressure, 1 point
8. Heart disease, 1 point
9. Circulation trouble in arms or legs, 1 point
10. Diabetes, 1 point
11. Stomach or intestinal disorders, 1 point
12. Osteoporosis, 1 point
13. Chronic liver or kidney disease, 1 point
14. Stroke, 1 point
15. Depression, 1 point

**eFigure 2:** Original response to single-item self-rated health question in our study cohort. Nearly half of the patients reported their overall health as being either fair (32%) or poor (14%).

**eFigure 3:** Receiver operating characteristic (ROC) curve for self-reported health for the diagnosis of frailty (panel A) and geriatric assessment (GA) identified impairments (panel B). Area under the curve for frailty is 0.83 and for GA impairment is 0.79. Dichotomizing SRH as good (good/very good/excellent) and poor (fair/poor) provided the best balance in terms of sensitivity and specificity.

**B**

**A**

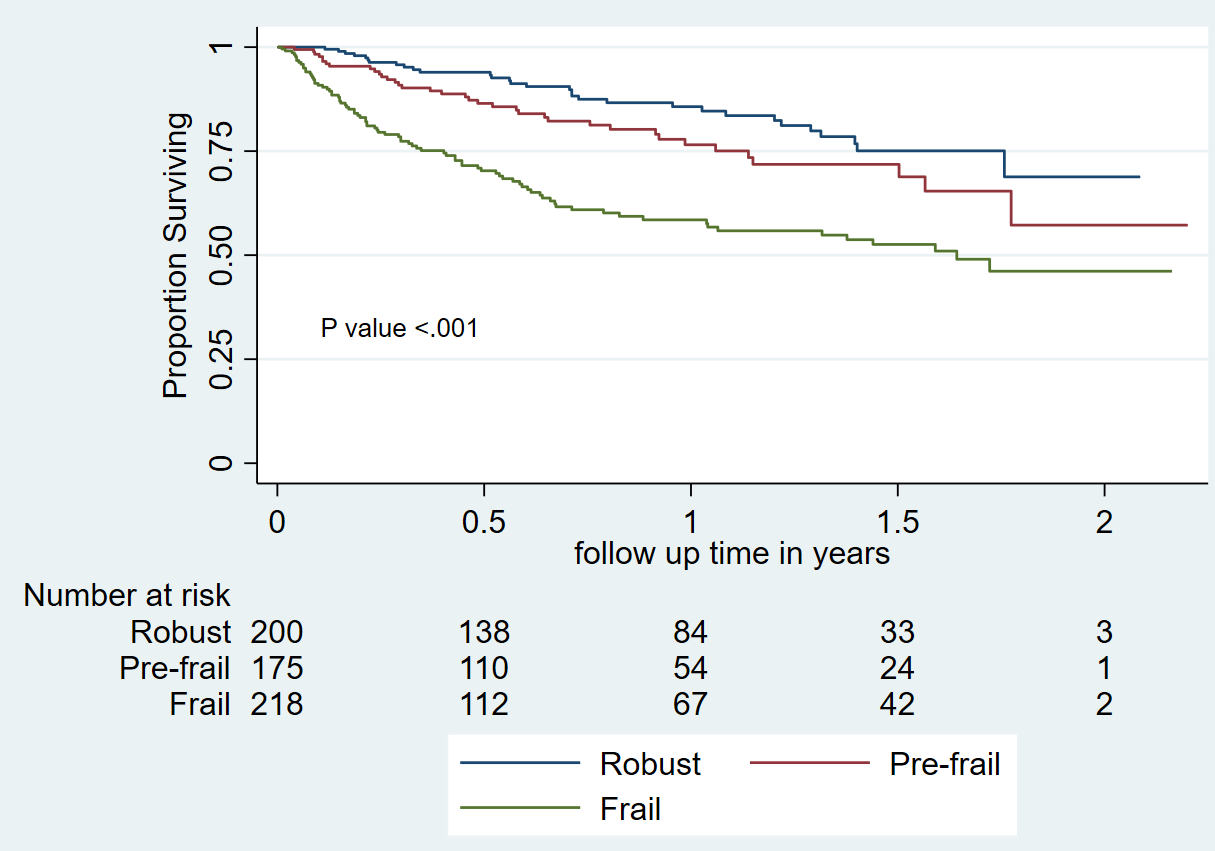


**eFigure 4:** Kaplan Meier survival plot showing the impact of frailty on overall survival. The 1-year survival among robust, pre-frail and frail patients was 85.7%, 76.5% and 58.5% respectively. The log-rank test for trend suggested that the survival distributions were statistically significant (*P*_trend_ <.001)

**eTable 2: Sensitivity Analysis for Evaluating Diagnostic Performance of Self Rated Health (SRH) for Identifying GA Impairment with Varying Cutoffs.**

| **Reference Standard** | **% Impaired** | **Sensitivity** | **Specificity** | **PPV** | **NPV** | **AUC (95% CI)** |
| --- | --- | --- | --- | --- | --- | --- |
| **Derivation Cohort (UAB CARE Registry)** | | | | | | |
| ≥1 GA impairment | 71.7% | 58.4% | 86.9% | 91.9% | 45.2% | 0.73 (0.69-0.76) |
| ≥3 GA impairment | 28.0% | 81.3% | 69.4% | 50.8% | 90.5% | 0.75 (0.72-0.79) |
| Age ≥70y & ≥2 GA impairment | 44.2% | 67.5% | 79.7% | 72.5% | 75.6% | 0.74 (0.68-0.79) |
| Age ≥70y & Frailty | 36.7% | 77.8% | 81.9% | 71.3% | 86.4% | 0.79 (0.75-0.85) |
| **Validation Cohort (University of North Carolina)** | | | | | | |
| ≥1 GA impairment | 55.8% | 41.7% | 92.8% | 87.9% | 55.7% | 0.67 (0.63-0.71) |
| ≥3 GA impairment | 11.5% | 63.2% | 80.1% | 29.3% | 94.4% | 0.72 (0.64-0.80) |
| Age ≥70y & ≥2 GA impairment | 26.6% | 59.3% | 88.7% | 65.3% | 85.9% | 0.74 (0.67-0.81) |
| Age ≥70y & Frailty | 17.8% | 53.4% | 83.2% | 40.7% | 89.3% | 0.68 (0.60-0.77) |

GA, geriatric assessment; PPV, positive predictive value; NPV, negative predictive value; AUC, area under the curve

**eTable 3: Multivariate Cox Proportional Hazards Regression Model showing Predictors of Overall Survival after Adjusting for Frailty.**

| **Variable** | **Hazard Ratio** | **95% CI** | ***P* value** |
| --- | --- | --- | --- |
| Self-Reported Health  - Good  - Poor | Ref  1.44 | 0.91-2.29 | .12 |
| Frailty Category  - Robust  - Pre-frail  - Frail | Ref  1.34  2.25 | -  0.79-2.27  1.27-3.98 | .28  .005 |
| Age (continuous) | 1.00 | 0.98-1.03 | .98 |
| Gender  - Female  - Male | Ref  1.05 | 0.74-1.48 | .79 |
| Race  - White/Caucasian  - Black/African American  - Other | Ref  0.83  2.98 | -  0.56-1.25  0.89-9.98 | .36  .14 |
| Planned Chemotherapy | 0.95 | 0.64-1.43 | .83 |
| Cancer Stage  - Stage I-II  - Stage III  - Stage IV | Ref  0.87  2.48 | 0.50-1.51  1.63-3.77 | .62  <.001 |

Cancer Type treated as a stratification variable

S1. Mediation Analysis methodology and main results:

We hypothesized that frailty might act as a mediator between SRH and overall survival. To test for this hypothesis, we used the four way decomposition as proposed by Vanderweele to conduct this mediation analysis. We estimated mediation with “exposure” defined as a dichotomized measure of self-reported health (poor/fair vs. good/excellent) and the mediator focused on frailty index as a continuous variable and fixing the level at its mean for the estimation of controlled direct effect. In the below table we have included the results of the four way decomposition mediation analysis, where the effect sizes are included in the ratio scale, and the total excess risk is broken down into four parts: a) excess relative risk due controlled direct effect, controlling the frailty index at its mean b) excess relative risk due to reference interaction (interaction only) c) excess relative risk due to mediated interaction (due to both interaction and mediation) and d) excess relative risk due to pure indirect effect. A total excess relative risk of -0.61 in the mean survival ratio scale indicates that as compared to those with good SRH, patients with poor SRH had a 61% lower mean survival time. On four-way decomposition, we found that of this total effect, pure mediation effect had an excess relative risk of -0.43, leading to the conclusion that frailty accounts for 69% (-0.43/-0.61) of the observed impact on survival.

**eTable4:** Output of the mediation analysis with causal effects estimated for a change in the exposure from good to poor Self Rated Health and at the mean level of all the covariates. Controlled direct effect computed fixing Frailty Index as a continuous variable at its mean.

| Variable | Coefficient | 95% Confidence Interval | P value |
| --- | --- | --- | --- |
| Total Excess Relative Risk | -0.62 | -0.76 to -0.48 | <.001 |
| a. Excess Relative Risk due to controlled direct effect | -0.95 | -1.64 to -0.27 | .01 |
| b. Excess relative risk due to reference interaction | 0.42 | -0.13 to 0.97 | .14 |
| c. Excess relative risk due to mediated interaction | 0.35 | -0.01 to 0.70 | .06 |
| d. Excess related risk due to pure indirect effect | -0.43 | -0.74 to -0.11 | .01 |

*model adjusted for age, sex, race/ethnicity, cancer type, cancer stage and planned chemotherapy.

**References:**

1. Fillenbaum GG, Smyer MA. The development, validity, and reliability of the OARS multidimensional functional assessment questionnaire. *Journals of Gerontology.* 1981;36(4):428-434.

2. Bauer J, Capra S, Ferguson M. Use of the scored Patient-Generated Subjective Global Assessment (PG-SGA) as a nutrition assessment tool in patients with cancer. *Eur J Clin Nutr.* 2002;56(8):779-785.

3. Teno J, Kiel D, Mor V. Multiple stumbles: a risk factor for falls in community-dwelling elderly. A prospective study. *J Am Geriatr Soc.* 1990;38(12):1321-1325.

4. Vigano AL, di Tomasso J, Kilgour RD, et al. The abridged patient-generated subjective global assessment is a useful tool for early detection and characterization of cancer cachexia. *J Acad Nutr Diet.* 2014;114(7):1088-1098.

5. Saffer BY, Lanting SC, Koehle MS, Klonsky ED, Iverson GL. Assessing cognitive impairment using PROMIS((R)) applied cognition-abilities scales in a medical outpatient sample. *Psychiatry Res.* 2015;226(1):169-172.

6. Pilkonis PA, Choi SW, Reise SP, et al. Item banks for measuring emotional distress from the Patient-Reported Outcomes Measurement Information System (PROMIS(R)): depression, anxiety, and anger. *Assessment.* 2011;18(3):263-283.

7. Riley WT, Pilkonis P, Cella D. Application of the National Institutes of Health Patient-reported Outcome Measurement Information System (PROMIS) to mental health research. *J Ment Health Policy Econ.* 2011;14(4):201-208.

8. Moser A, Stuck AE, Silliman RA, Ganz PA, Clough-Gorr KM. The eight-item modified Medical Outcomes Study Social Support Survey: psychometric evaluation showed excellent performance. *J Clin Epidemiol.* 2012;65(10):1107-1116.

9. Lees J, Chan A. Polypharmacy in elderly patients with cancer: clinical implications and management. *The Lancet Oncol.* 2011;12(13):1249-1257.

10. Klepin HD, Pitcher BN, Ballman KV, et al. Comorbidity, chemotherapy toxicity, and outcomes among older women receiving adjuvant chemotherapy for breast cancer on a clinical trial: CALGB 49907 and CALGB 361004 (alliance). *Journal of oncology practice.* 2014;10(5):e285-292.

11. Hays RD, Bjorner JB, Revicki DA, Spritzer KL, Cella D. Development of physical and mental health summary scores from the patient-reported outcomes measurement information system (PROMIS) global items. *Quality of life research : an international journal of quality of life aspects of treatment, care and rehabilitation.* 2009;18(7):873-880.

12. Rockwood K, Mitnitski A. Frailty in relation to the accumulation of deficits. *J Gerontol A Biol Sci Med Sci.* 2007;62:722-727.

13. Searle SD, Mitnitski A, Gahbauer EA, Gill TM, Rockwood K. A standard procedure for creating a frailty index. *BMC Geriatr.* 2008;8:24-24.

14. Guerard EJ, Deal AM, Chang Y, et al. Frailty Index Developed From a Cancer-Specific Geriatric Assessment and the Association With Mortality Among Older Adults With Cancer. *J Natl Compr Canc Netw.* 2017;15(7):894-902.

15. Cohen HJ, Smith D, Sun C-L, et al. Frailty as determined by a comprehensive geriatric assessment-derived deficit-accumulation index in older patients with cancer who receive chemotherapy. *Cancer.* 2016;122(24):3865-3872.

16. Mitnitski A, Song X, Skoog I, et al. Relative fitness and frailty of elderly men and women in developed countries and their relationship with mortality. *J Am Geriatr Soc.* 2005;53(12):2184-2189.
